# Amodal completion across the brain: The impact of structure and knowledge

**DOI:** 10.1167/jov.24.6.10

**Published:** 2024-06-13

**Authors:** Jordy Thielen, Tessa M. van Leeuwen, Simon J. Hazenberg, Anna Z. L. Wester, Floris P. de Lange, Rob van Lier

**Affiliations:** 1 Donders Institute for Brain, Cognition and Behaviour, Radboud University, Nijmegen, the Netherlands; 2 Department of Communication and Cognition, Tilburg University, Tilburg, the Netherlands; 3 Donders Institute for Brain, Cognition and Behaviour, Radboud University, Nijmegen, the Netherlands; 4 Laboratory for Biological Psychology, KU Leuven, Leuven, Belgium Donders Institute for Brain, Cognition and Behaviour, Radboud University, Nijmegen, the Netherlands; 5 Donders Institute for Brain, Cognition and Behaviour, Radboud University, Nijmegen, the Netherlands; 6 Donders Institute for Brain, Cognition and Behaviour, Radboud University, Nijmegen, the Netherlands; 7 Donders Institute for Brain, Cognition and Behaviour, Radboud University, Nijmegen, the Netherlands

**Keywords:** amodal completion, functional magnetic resonance imaging, expectation suppression, knowledge, repetition suppression, structure

## Abstract

This study investigates the phenomenon of amodal completion within the context of naturalistic objects, employing a repetition suppression paradigm to disentangle the influence of structure and knowledge cues on how objects are completed. The research focuses on early visual cortex (EVC) and lateral occipital complex (LOC), shedding light on how these brain regions respond to different completion scenarios. In LOC, we observed suppressed responses to structure and knowledge-compatible stimuli, providing evidence that both cues influence neural processing in higher-level visual areas. However, in EVC, we did not find evidence for differential responses to completions compatible or incompatible with either structural or knowledge-based expectations. Together, our findings suggest that the interplay between structure and knowledge cues in amodal completion predominantly impacts higher-level visual processing, with less pronounced effects on the early visual cortex. This study contributes to our understanding of the complex mechanisms underlying visual perception and highlights the distinct roles played by different brain regions in amodal completion.

## Introduction

To understand and navigate our intricate surroundings in this complex world, we rely on the wealth of information provided by our senses. However, at the same time, sensory input alone does not suffice to understand and navigate the world. Visual perception, despite seeming like a swift and effortless process, is in fact a highly complex process, with numerous factors contributing to its challenging nature. One such factor is the fragmented and incomplete nature of the retinal projection, as multiple objects enter the eye, potentially (partially) occluding one another or parts of themselves. However, in our visual experiences, these fragmented and incomplete objects are rarely perceived as such. Instead, they are seamlessly interpreted as continuous, whole entities within a vivid and three-dimensional world. This study aims to investigate the degree to which such perceptual completion relies on various cues, including low-level stimulus-driven automatic bottom-up factors like linear continuation of contours, and high-level knowledge-driven cognitive top-down factors like object familiarity. Additionally, this study seeks to identify the specific brain regions involved throughout this process.

Perceptual completion can be regarded as a form of perceptual filling in, which involves the experience of interpolation, sensing that missing information is being seamlessly completed (Komatsu, 2006; van Lier & Gerbino, 2015; Gerbino, 2020). Among the various forms of perceptual filling-in, one prominent type is amodal completion. Amodal completion entails perceiving occluded objects as consistent and continuous across space and time, even in the absence of a visual experience of the occluded parts themselves (Michotte, Thinès, & Crabbé, 1964, 1991). Amodal completion is often contrasted with modal completion, another perceptual completion process characterized by the subjective perception of illusory contours, typically occupying the foreground (Michotte & Burke, 1951; Kanizsa, 1976). Given the fact that amodal completion is not accompanied with illusory contours or surfaces, one might intuitively think that a variety of sources might be utilized in order to fill in missing parts and that this is not limited to low-level stimulus-driven factors. Still, to what extent amodal completion is purely stimulus driven and cognitively impenetrable remains a topic of discussion.

Several studies have indeed argued that amodal completion operates as a purely stimulus-driven automatic bottom-up process and remains resistant to knowledge-driven cognitive top-down influences (Kanizsa, 1979). This notion is supported by various demonstrations, such as the famous Michotte triangle, which illustrates that amodal completion follows its own course regardless of cognitive efforts (Michotte, Thinès, & Crabbé, 1991). Additionally, magicians often exploit amodal presence (Ekroll, Sayim, & Wagemans, 2013; Ekroll, Sayim, van der Hallen, & Wagemans, 2016; Ekroll & Wagemans, 2016) and amodal absence (Ekroll, Sayim, & Wagemans, 2017; Ekroll, de Bruyckere, Vanwezemael, & Wagemans, 2018; Svalebjørg, Øhrn, & Ekroll, 2020; Ekroll et al., 2021) in their tricks, because studies have shown that magic tricks relying on amodal completion are more challenging to decipher after repeated exposure compared to those based on other cues, such as diverting attention, showing the resistance to cognitive influences (Ekroll, Mertens, & Wagemans, 2018).

In accordance with the stimulus-driven account, the perceived phenomenon of amodal completion has prompted the development of several theoretical explanations, which can be categorized as either “local” or “global” accounts. Local accounts emphasize the influence of local cues that guide the completion process, aligning with the principles of the Gestalt law of good continuation. According to this law, elements forming a line or curve appear more related than randomly positioned elements (Koffka, 1935). Smooth contours originating from these junctions are more likely to be perceived as continuous compared to contours with sharp angles (Kanizsa, 1975; Kanizsa, 1979; Kanizsa, 1985). The “relatability criterion” formalizes this theory, suggesting that disconnected edges (e.g., due to occlusion) can be connected if there exists a smooth and monotonic interpolation (Kellman & Shipley, 1991; Kellman, 2003).

In contrast to local accounts, global accounts propose that completion processes involve regularities such as symmetries. Global theories align with the Gestalt law of Prägnanz, which suggests that ambiguous elements are interpreted in the simplest way possible, with fewer elements and symmetrical configurations favored over asymmetrical ones (Koffka, 1935). It has been argued that amodal completion tends to favor configurations that require the least amount of information to describe (Hochberg & McAlister, 1953). This idea was further formalized in the structural information theory (SIT), which quantifies the information contained in visual patterns (Leeuwenberg, 1969; Leeuwenberg, 1971). Indeed, in line with SIT, the most preferred completions are typically the simplest and most regular ones (Buffart, Leeuwenberg, & Restle, 1981). While many studies have demonstrated a dominance of global cues (Sekuler, Palmer, & Flynn, 1994; Sekuler, 1994; van Lier, Leeuwenberg, & van der Helm, 1995; van Lier & Wagemans, 1999), others have presented examples in which completion processes deviate from regularity (Rock, 1983; Boselie, 1988; Boselie & Wouterlood, 1992).

In conflict with these two stimulus-driven accounts, amodal completion seems not solely influenced by low-level stimulus configurations but also impacted by various high-level contextual cues, as demonstrated in several studies. These cues encompass factors such as lighting conditions (Kim, Jeng, & Anderson, 2014), spatial context (Rauschenberger, Peterson, Mosca, & Bruno, 2004), temporal context (Plomp & van Leeuwen, 2006), task demands (Lee & Vecera, 2005), and familiarity with material properties (Gerbino & Zabai, 2003; Vrins, de Wit, & van Lier, 2009) and object shapes (Hazenberg & van Lier, 2016; Yun, Hazenberg, & van Lier, 2018). Moreover, research has shown that explicit learning can influence the preferred completion for occlusion patterns, especially in cases where local and global cues conflict (Hazenberg, Jongsma, Koning, & van Lier, 2014).

The extent to which amodal completion relies exclusively on bottom-up factors versus the potential contribution of top-down mechanisms remains a topic of considerable uncertainty. This inquiry is intrinsically linked to the neural dynamics of amodal completion (for a review, see Thielen, Bosch, van Leeuwen, van Gerven, & van Lier, 2019b). Are low-level visual areas actively recruited in the completion process, particularly in completions that rely more on stimulus-driven factors? Alternatively, does the early visual cortex merely encode the fragmented, mosaic-like stimulus initially captured by the retinal image, to undergo subsequent refinement into a holistic depiction of the entire object within higher-level visual areas?

A limited number of primate studies have observed activity in the early visual cortex responding to partially occluded objects (Sugita, 1999; Bakin, Nakayama, & Gilbert, 2000; Lee & Nguyen, 2001; Bushnell, Harding, Kosai, & Pasupathy, 2011; Kosai, El-Shamayleh, Fyall, & Pasupathy, 2014; Fyall, El-Shamayleh, Choi, Shea-Brown, & Pasupathy, 2017). For example, Sugita (1999) identified V1 neurons responding to invisible line segments caused by occlusion. In investigations involving human participants, some studies documented early visual cortex activity (Rauschenberger, Liu, Slotnick, & Yantis, 2006; Hulme & Zeki, 2007; Ban et al., 2013; Erlikhman & Caplovitz, 2017; de Haas & Schwarzkopf, 2018), while others reported a lack of such activity (Lerner, Hendler, & Malach, 2002; Lerner, Harel, & Malach, 2004; Olson, Gatenby, Leung, Skudlarski, & Gore, 2004; Weigelt, Singer, & Muckli, 2007).

Notably, Rauschenberger et al. (2004), using a functional magnetic resonance imaging (fMRI) repetition suppression paradigm, demonstrated that early visual areas exclusively represented the physically visible parts of the occluded object up to 100 ms, but after 250 ms, they began to encode the completed object. However, a subsequent study employing a similar repetition suppression paradigm failed to identify a completed representation in the early visual cortex even after 300 ms (Weigelt et al., 2007). Consequently, the extent to which the early visual cortex represents the completed object remains uncertain.

Concerning higher-order visual areas, particularly the lateral occipital complex (LOC), recognized for its involvement in object recognition (Kourtzi & Kanwisher, 2001), the findings exhibit greater consistency. Numerous studies consistently report LOC activity in response to partially occluded objects (Yin, Shimojo, Moore, & Engel, 2002; Lerner et al., 2002; Lerner et al., 2004; Rauschenberger et al., 2006; Weigelt et al., 2007; Hulme & Zeki, 2007; Hegdé, Fang, Murray, & Kersten, 2008; Erlikhman & Caplovitz, 2017). Moreover, magnetoencephalography (MEG) observations include a mismatch negativity (MMN) in response to local and global completions (de Wit, Bauer, Oostenveld, Fries, & van Lier, 2006).

**Figure 1.**
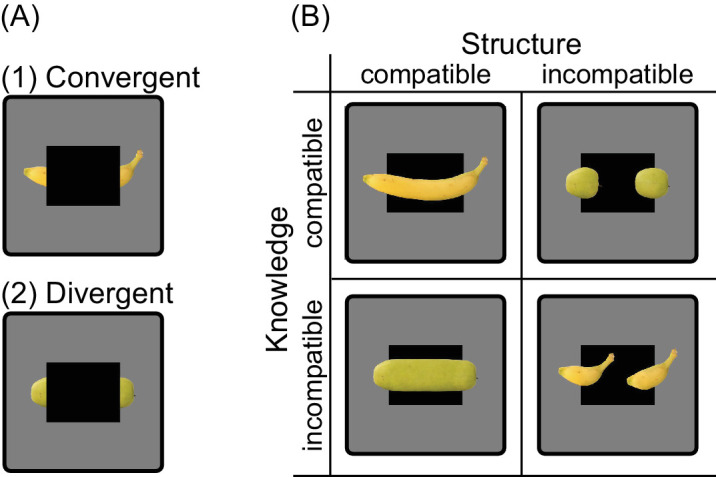
Convergent and divergent stimuli. (A) In this study, there were two types of partially occluded stimuli. For convergent stimuli (A1, e.g., here a banana), compatibility with structural cues and knowledge align to yield the same inferred shape. Conversely, for divergent stimuli (A2, e.g., here an apple), compatibility with structural cues and knowledge lead to different interpretations, resulting in distinct inferred shapes. (B) Specifically, completions may exhibit compatibility (S+), as shown in the left column, or incompatibility (S−) with the underlying structural cues, depicted in the right column. Furthermore, these completions may either align with knowledge (K+), as seen in the upper row, or clash with it (K−), as illustrated in the lower row. Adapted from Hazenberg and van Lier (2016).

Significantly, Hazenberg and van Lier (2016) utilized well-known naturalistic objects to investigate the influence of both low-level stimulus properties and high-level object familiarity on amodal completion, employing electroencephalography (EEG) in their study. The presented objects included convergent completions that were either consistent or inconsistent with both low-level stimulus-driven cues and high-level knowledge cues, as well as divergent completions aligning with one cue while conflicting with the other (see Figure 1). Their study revealed a late P3 event-related potential (ERP) with amplitude variations corresponding to completions triggered by both stimulus-driven and knowledge cues. These ERP responses were localized around parieto-occipital regions, potentially associated with LOC activity, and show that amodal completion does not solely rely on stimulus structure but also on object familiarity.

This study seeks to investigate the involvement of both low-level and higher-level visual areas in amodal completion, exploring whether their activation is driven by bottom-up structural cues (e.g., linear continuations) or top-down knowledge cues (e.g., object familiarity). Building upon the foundational work of Rauschenberger et al. (2006) and Weigelt et al. (2007), which utilized line-drawing abstract shapes, our research introduces naturalistic stimuli, specifically, the fruit and vegetable stimuli from the EEG study conducted by Hazenberg and van Lier (2016). This stimulus set provides a unique opportunity to disentangle the processes of more automatic stimulus-driven completions and completions influenced by high-level expectations. We build on the work of Hazenberg et al. (2014) by employing an fMRI repetition suppression paradigm, enabling us to verify the ERP results and pinpoint the exact brain regions that are involved in representing completions based on different structure and knowledge cues.

## Methods

### Participants

We opted for a minimum sample size of *N* = 34 participants to ensure β = 80% power for detecting a medium effect size (Cohen’s *d* ⩽ 0.5) using a two-sided paired *t*-test at a significance level of α = 0.05. In total, 40 healthy right-handed participants were recruited from the Radboud University research participation system. Four of these participants took part in pilot experiments and were not included in the analysis. Two participants did not complete the full experimental protocol and were excluded from the analysis. The resulting *N* = 34 participants (21 females and 13 males, age 19–30 years with μ = 23 and σ = 2 years) reported no history of epilepsy or claustrophobia, had normal or corrected-to-normal vision, and reported no central nervous system abnormalities. All participants gave written informed consent prior to the experiment and received payment after the experiment. The experimental procedure and methods were approved by and performed in accordance with the guidelines of the local ethics committee (CMO region Arnhem-Nijmegen, the Netherlands).

### Equipment

Anatomical and fMRI images were acquired on a 3T Skyra scanner (Siemens, Erlangen, Germany), using a 32-channel head coil. Anatomical images were acquired using a T1-weighted magnetization prepared rapid gradient echo sequence (MPRAGE) in the sagittal orientation (repetition time (TR) = 2,300 ms, echo time (TE) = 3.03 ms, 8 degree flip angle, field of view (FOV) = 256 mm, voxel size 1 mm isotropic, generalized autocalibrating partial parallel acquisition (GRAPPA) acceleration factor = 2). Functional images were acquired using a whole-brain T2*-weighted multiband-4 sequence (TR = 1,500 ms, TE = 39.6 ms, 75 degree flip angle, FOV = 210 mm, 68 slices, voxel size 2 mm isotropic) with an anterior to posterior phase-encoding gradient.

An Eyelink 1000 Plus (SR Research, Ltd., Ottawa, Canada) was used to record monocular eye movements and pupil dilation of the left eye. To avoid vibrations caused by the MR scanner, the Eyelink camera was attached to a dedicated ceiling mount. The Eyelink camera tracked the left eye at 500 Hz via a mirror mounted on the head coil and was calibrated at the start of a session using using a 9-point calibration procedure. Eye tracking was performed for a post hoc analysis of fixation accuracy and to rule out any potential confounding eye movement effects (Thielen, van Lier, & van Gerven, 2018; Thielen, Bosch, van Leeuwen, van Gerven, & van Lier, 2019a).

Stimuli were presented on a 32-in. MR-compatible IPS LCD with 1,920-pixel × 1,080-pixel resolution (698.4×392.9 mm), 120 Hz refresh rate, and brightness of 80 cd/m^2^. The screen was visible using an adjustable mirror mounted on the head coil approximately 10 cm away from the participant’s eye. The screen was placed at the end of the scanner bore with an approximate distance of 1,086 mm to the participant at iso-center.

### Stimuli

**Figure 2.**
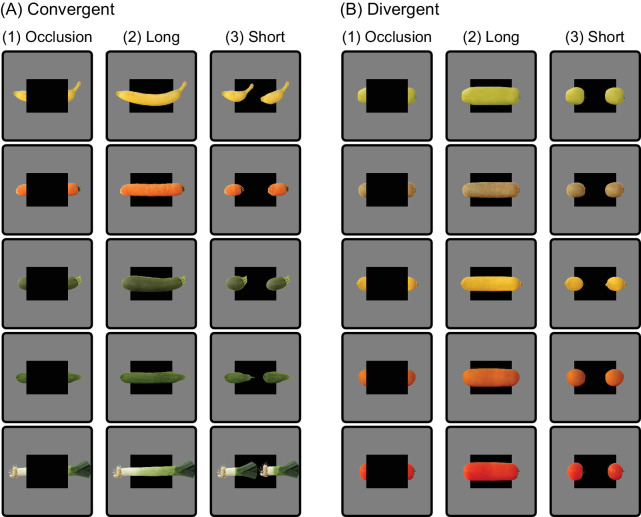
Fruit and vegetable stimuli. Ten different fruits and vegetables were used as stimuli, of which five were convergent (A) and five were divergent (B). Convergent stimuli were a banana, carrot, zucchini, cucumber, and leek and were those of which the occlusion stimulus (A1) could be completed either compatible with both structure as well as knowledge (A2) or both incompatible with structure and knowledge (A3). Divergent stimuli were an apple, kiwi, lemon, orange, and tomato and were those of which the occlusion (B1) could be completed either compatible with structure but incompatible with knowledge (B2) or incompatible with structure but compatible with knowledge (B3). Adapted from Hazenberg and van Lier (2016).

Stimuli were naturalistic, full-color images of fruits and vegetables as used in the study of Hazenberg and van Lier (2016). The stimulus set contained 10 different well-known fruits and vegetables (see Figure 2): banana, carrot, zucchini, cucumber, leek, apple, kiwi, lemon, orange, and tomato. The stimulus set contained two types of stimuli: Convergent stimuli (Figure 2A) carried structure and knowledge cues converging to the same shape, while divergent stimuli (Figure 2B) carried structure and knowledge cues that each led to a different shape. Three versions were created for each of the stimuli: (1) an occlusion version (Figure 2A1/B1), a one-piece long version (Figure 2A2/B2), and a two-piece short version (Figure 2A3/B3). Stimuli were always presented on a mean-luminance gray background and subtended a maximum of 7.0 degrees of visual angle horizontally. At the center of the screen, overlaid on top of the stimuli, a fixation cross was presented that subtended 0.25 degrees of visual angle. Finally, the stimuli always contained a black rectangle that was either in the foreground, partially occluding the center of the objects (i.e., occlusion versions), or in the background, completely revealing the objects (i.e., the short and long versions).

### Procedure

Participants completed the experiment in one single session, which started with a localizer, head-scout, and fieldmap (approximately 10 min). Subsequently, the first half of the main task was presented (approximately 20 min), after which the anatomical scan was performed (approximately 10 min), followed by the remaining half of the main task (approximately 20 min). Finally, participants completed a single LOC localizer run (approximately 18 min) and a single retinotopy run (approximately 12 min).

The stimulus presentation was performed using *Python* 3.7.10 and *PsychoPy* 2021.2.0 (Peirce et al., 2019).

#### Main task

Participants were engaged in a total of 480 experimental trials throughout the study. For 6 of the 34 participants, the experimental trials were split into two runs: one before and one after the anatomical scan. Each of these runs consisted of 240 experimental trials. Following these initial six participants, the remaining participants had their experimental trials divided into four runs to accommodate two additional short breaks. These four runs included two runs before and two runs after the anatomical scan, each consisting of 120 experimental trials. Each run began and ended with a 6-s period of pure fixation.

**Figure 3.**
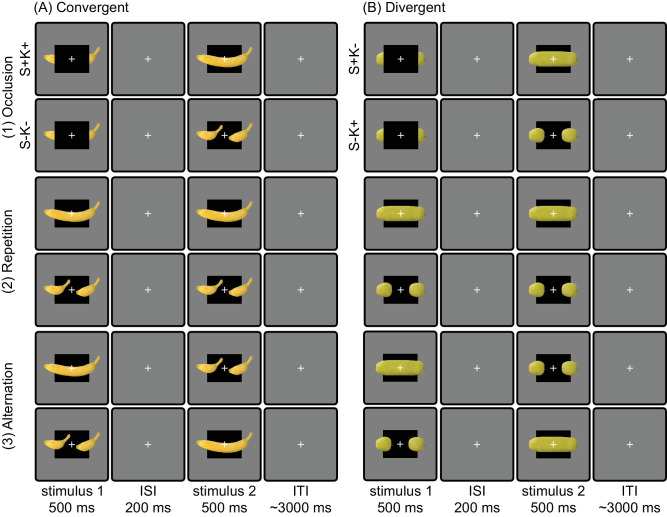
Experimental conditions and timeline. In the main task of the experiment, the trials consisted of pairs of stimuli featuring a single kind of fruit or vegetable. Trials presented a pair of convergent (A) or divergent (B) stimuli that involved an (1) occlusion, (2) repetition, or (3) alternation. This resulted in a total of 12 conditions. Each of these conditions was presented in one of four configurations, accounting for all possible horizontally and vertically mirrored versions. Finally, for each of two stimulus types (convergent or divergent), there were five different fruits or vegetables; see Figure 2. Importantly, the occlusion trials feature the four main conditions of the experiment, including all combinations of compatibility (+) or incompatibility (−) with the structure (S) and knowledge (K) cues. All trials presented an initial stimulus for 500 ms, following an interstimulus interval (ISI) of 200 ms, a second stimulus for 500 ms, and finally an intertrial interval (ITI) of on average 3,000 ms.

The experimental trials involved presenting the participants with two different stimulus types (convergent or divergent) featuring five different fruits or vegetables (see Figure 2), encompassing six different conditions (two occlusion, two repetition, two alternation; see Figure 3) and four different orientations (vertically and horizontally mirrored). Each combination of stimulus type, fruit or vegetable, condition, and orientation was repeated twice, resulting in a total of 480 experimental trials.

During a trial, a pair of stimuli was presented featuring the same fruit or vegetable of the same orientation, with the first stimulus displayed for 500 ms, followed by a 200-ms interstimulus interval, and then the second stimulus presented for 500 ms (see Figure 3). The intertrial time varied and was determined by sampling from a truncated exponential distribution with a minimum of 2 s, a mean of 3 s, and a maximum of 10 s. The selection of a 500-ms stimulus time has been proven effective in revealing noticeable effects of knowledge on amodal completion (Yun et al., 2018). Likewise, a 200-ms interstimulus interval has been shown to optimize the repetition suppression effect throughout visual cortex (Fritsche, Lawrence, & de Lange, 2020). These pairings generated six distinct conditions for each stimulus type (convergent and divergent): two occlusion trials, two repetition trials, and two alternation trials (see Figure 3).

In repetition trials (Figure 3A2/B2), both stimuli shared the same shape (e.g., both were long), while in alternation trials (Figure 3A3/B3), the first stimulus featured one shape (e.g., short), while the second stimulus featured the other shape (e.g., long). Note, in each of these stimulus presentations, the black occluder was always present in the background. According to the classic repetition suppression effect, the blood oxygen level dependent (BOLD) response to a repetition trial should be suppressed relative to an alternation trial (Henson & Rugg, 2003).

Of interest, in occlusion trials, the completion could either be compatible with linear continuation of contours and/or could be compatible with knowledge, which can be studied by investigating their relative suppression effects. For convergent stimuli, the two occlusion trials (Figure 3A1) are labeled as structure and knowledge compatible (S+K+) if the occlusion stimulus is followed by a long shape and structure and knowledge incompatible (S−K−) if it is followed by a short stimulus. For divergent stimuli, the occlusion trials (Figure 3B1) are denoted as structure compatible but knowledge incompatible (S+K−) if the occlusion is followed by a long shape, and structure incompatible but knowledge compatible (S−K+) if it is followed by a short shape. We hypothesized that the BOLD response to a surprising occlusion trial, such as structure and knowledge incompatible (S−K−), will be larger than the BOLD response to a familiar one, such as structure and knowledge compatible (S+K+). Interestingly, the BOLD response to an occlusion trial would be increased or dampened depending on whether an area is sensitive to a structural and/or knowledge cue in the divergent cases where one cue is compatible and the other incompatible (S+K−, S−K+).

The order of trials and the intertrial intervals were pseudo-randomized using AFNI’s make_random_timing.py. A total of 100,000 random experimental designs were simulated, and the top 40 designs were selected based on the explained variance of various contrasts of interest.

Throughout the experimental trials, participants were instructed to maintain fixation on the fixation cross. As a means to maintain and to check the participant’s attention on the stimuli, in between the experimental trials, 16 task trials were randomly interspersed, during which a 1-back task was performed. With a probability of 0.5, participants encountered a statement regarding either the first or second stimulus presented in the preceding trial. The statement asserted whether the image depicted a short or long fruit or vegetable. In these statements, either the shape of the object or the object itself could be incorrect. If the statement was determined to be correct (with a 0.5 probability), participants were required to press Button 1 on a button box using their right index finger. Conversely, if the statement was incorrect, participants pressed Button 2 using their right middle finger. Participants received immediate feedback on the correctness of their answer by coloring the fixation cross green (correct) or red (incorrect).

#### Functional localizer

Following the experimental runs, participants proceeded to perform a single localizer run. The purpose of this run was to identify the object-selective LOC specifically for the fruit and vegetable stimuli. The information obtained from this run would be utilized to define participant-specific functional region of interest (ROI) masks, which would help select the most informative voxels for further analysis.

The localizer run employed a block design consisting of repeated presentations of the original fruit and vegetable stimuli without any occluder (not even in the background), as well as their phase-scrambled counterparts. The run commenced and concluded with 6 s of pure fixation. In between, a total of 96 localizer trials were presented, with 7 randomly interspersed blank trials. These localizer trials encompassed two conditions (original or scrambled), two stimulus types (convergent or divergent), and two stimulus shapes (long or short), each repeated 12 times. Unlike the experimental trials, the localizer trials included sequences of mixed fruits and vegetables, but only those that belonged to the same stimulus type (convergent, divergent) and shared the same stimulus shape (long, short).

Each trial lasted for 10 s and featured stimuli presented at a frequency of 2 Hz (300 ms on and 200 ms off). Within a trial, all stimuli of a specific type (convergent or divergent) and a single stimulus shape (long or short) were presented in each of their different orientations. These 20 stimuli were displayed in a randomized order without an intertrial interval. The sequence of trials was fully randomized within the run.

During the localizer run, participants were instructed to maintain fixation on the fixation cross. Approximately once every 20 s, the brightness of the fixation cross would dim, prompting participants to press Button 1 using their right index finger in response.

#### Retinotopy

Following the localizer run, participants finally performed a single retinotopy run. Due to time constraints, 5 of 34 participants did not complete this retinotopy run. This run, however, is not used in the current study.

The retinotopy run commenced and concluded with 6 s of pure fixation. Within the run, a bar stimulus traversed the screen in eight different directions, with each direction repeated twice. The order of trials was fixed for all participants. The width of the bar subtended 1.5 degrees of visual angle and was overlaid with a full-contrast black and white checkerboard pattern featuring a spatial frequency of 1 cycle per visual degree. The bar stimulus was presented within an annulus covering 8 degrees of visual angle.

Each trial within the retinotopy run lasted for 30 s without any intertrial intervals. However, after every two directions, a blank 30-s trial was inserted. During a bar trial, the bar would traverse from one end of the annulus to the other end in 20 steps of 1.5 s each. Throughout the trial, the contrast of the bar changed at a frequency of 2 Hz (250 ms on and 250 ms off).

Similar to the localizer run, participants were instructed to maintain fixation on the fixation cross throughout the retinotopy run. Approximately once every 20 s, the brightness of the fixation cross would dim, signaling participants to press Button 1 using their right index finger in response.

### fMRI data preprocessing

All raw MRI data were converted to the brain imaging data structure (BIDS) (Gorgolewski et al., 2016) using *BIDScoin* 3.7.2 (Zwiers, Moia, & Oostenveld, 2022), including conversion to NiFTi format, and supplemented with standardized metadata.

Results included in this manuscript came from preprocessing performed using *fMRIPrep* 21.0.2 (Esteban, Blair et al., 2018; Esteban, Markiewicz et al., 2018; RRID:SCR_016216), which was based on *Nipype* 1.6.1 (Gorgolewski et al., 2011; Gorgolewski et al., 2018; RRID:SCR_002502). Many internal operations of *fMRIPrep* used *Nilearn* 0.8.1 (Abraham et al., 2014, RRID:SCR_001362), mostly within the functional processing workflow.

#### Anatomical data preprocessing

Per participant, the T1-weighted (T1w) image was corrected for intensity nonuniformity (INU) with N4BiasFieldCorrection (Tustison et al., 2010), distributed with ANTs 2.3.3 (Avants, Epstein, Grossman, & Gee, 2008, RRID:SCR_004757), and used as T1w reference throughout the workflow. The T1w reference was then skull-stripped with a *Nipype* implementation of the antsBrainExtraction.sh workflow (from ANTs), using OASIS30ANTs as the target template. Brain tissue segmentation of cerebrospinal fluid (CSF), white matter (WM), and gray matter (GM) was performed on the brain-extracted T1w using fast (FSL 6.0.5.1:57b01774, RRID:SCR_002823, Zhang, Brady, & Smith, 2001). Volume-based spatial normalization to one standard space (MNI152NLin2009cAsym) was performed through nonlinear registration with antsRegistration (ANTs 2.3.3), using brain-extracted versions of both T1w reference and the T1w template. The following template was selected for spatial normalization: *ICBM 152 Nonlinear Asymmetrical template version 2009c* (Fonov, Evans, McKinstry, Almli, & Collins, 2009; RRID:SCR_008796; TemplateFlow ID: MNI152NLin2009cAsym).

#### Functional data preprocessing

For each of the BOLD runs found per participant, the following preprocessing was performed. First, a reference volume and its skull-stripped version were generated using a custom methodology of *fMRIPrep*. Head-motion parameters with respect to the BOLD reference (transformation matrices and six corresponding rotation and translation parameters) were estimated before any spatiotemporal filtering using mcflirt (FSL 6.0.5.1:57b01774; Jenkinson, Bannister, Brady, & Smith, 2002). The BOLD time-series (no slice-timing correction was applied) were resampled onto their original, native space by applying the transforms to correct for head motion. These resampled BOLD time-series will be referred to as *preprocessed BOLD in original space*, or just *preprocessed BOLD*. The BOLD reference was then coregistered to the T1w reference using mri_coreg (FreeSurfer) followed by flirt (FSL 6.0.5.1:57b01774; Jenkinson & Smith, 2001) with the boundary-based registration (Greve & Fischl, 2009) cost function. Coregistration was configured with 6 degrees of freedom. Several confounding time-series were calculated based on the *preprocessed BOLD*: framewise displacement (FD), DVARS, and three region-wise global signals. FD was computed using two formulations following Power (absolute sum of relative motions; Power et al., 2014) and Jenkinson (relative root mean square displacement between affines; Jenkinson et al., 2002). FD and DVARS were calculated for each functional run, both using their implementations in *Nipype* (following the definitions by Power et al., 2014). The three global signals were extracted within the CSF, the WM, and the whole-brain masks. Additionally, a set of physiological regressors was extracted to allow for component-based noise correction (*CompCor*; Behzadi, Restom, Liau, & Liu, 2007). Principal components were estimated after high-pass filtering the *preprocessed BOLD* time-series (using a discrete cosine filter with 128-s cutoff) for the two *CompCor* variants: temporal (tCompCor) and anatomical (aCompCor). tCompCor components were then calculated from the top 2% variable voxels within the brain mask. For aCompCor, three probabilistic masks (CSF, WM, and combined CSF+WM) were generated in anatomical space. The implementation differed from that of Behzadi et al. (2007) in that instead of eroding the masks by 2 pixels on BOLD space, the aCompCor masks were subtracted a mask of pixels that likely contained a volume fraction of GM. This mask was obtained by thresholding the corresponding partial volume map at 0.05, and it ensured components were not extracted from voxels containing a minimal fraction of GM. Finally, these masks were resampled into BOLD space and binarized by thresholding at 0.99 (as in the original implementation). Components were also calculated separately within the WM and CSF masks. For each CompCor decomposition, the *k* components with the largest singular values were retained, such that the retained components’ time series were sufficient to explain 50% of variance across the nuisance mask (CSF, WM, combined, or temporal). The remaining components were dropped from consideration. The head-motion estimates calculated in the correction step were also placed within the corresponding confounds file. The confound time series derived from head-motion estimates and global signals were expanded with the inclusion of temporal derivatives and quadratic terms for each (Satterthwaite et al., 2013). Frames that exceeded a threshold of 0.5 mm FD or 1.5 standardized DVARS were annotated as motion outliers. The BOLD time-series were resampled into standard space, generating a *preprocessed BOLD run in MNI152NLin2009cAsym space*. First, a reference volume and its skull-stripped version were generated using a custom methodology of *fMRIPrep*. All resamplings can be performed with *a single interpolation step* by composing all the pertinent transformations (i.e., head-motion transform matrices, susceptibility distortion correction when available, and coregistrations to anatomical and output spaces). Gridded (volumetric) resamplings were performed using antsApplyTransforms (ANTs), configured with Lanczos interpolation to minimize the smoothing effects of other kernels (Lanczos, 1964). Nongridded (surface) resamplings were performed using mri_vol2surf (FreeSurfer).

### fMRI ROI definition

The functional images of the localizer run underwent steps that involved spatial smoothing with SUSAN using a Gaussian kernel (full width at half maximum of 5 mm) and temporal high-pass filtering with a cutoff at 100 s. The localizer run was employed for a first-level analysis to identify participant-specific ROIs. For this analysis, two regressors were included, corresponding to the two stimulus types (original and scrambled). Additionally, the design matrix incorporated confound regressors calculated via fMRIPrep, including framewise displacement, six standard motion parameters, six anatomical CompCor components, two cosines, and motion outliers.

Using FSL FEAT, voxel-wise general linear models (GLMs) were fitted to each participant’s run data. The events were modeled as regressors with a duration of 10 s, capturing the combined duration of the 20 presented stimuli in a trial. The regressors were convolved with a double gamma hemodynamic response function.

Two ROIs were identified in the study: early visual cortex (EVC) and the LOC. LOC was defined based on its significant preference for intact versus scrambled objects, as previously demonstrated in the literature (Kourtzi & Kanwisher, 2001). Specifically, an LOC mask was created on an individual participant basis by selecting the significantly active voxels from the “original versus scrambled” contrast by thresholding *z* > =5 (uncorrected; that is, *p* < 1e − 6). If needed, the threshold was iteratively lowered until at least 200 voxels were in the mask. Additionally, the ROI mask was limited using an anatomical mask of the Harvard Oxford atlas, by removing voxels outside the lateral occipital cortex inferior division. The resulting LOC mask contained both ventral and lateral components, including LO1 and LO2 (Larsson & Heeger, 2006).

On the other hand, the EVC mask was generated on an individual participant basis, selecting the significantly active voxels from the “mean activation” contrast by thresholding *z* > =5 (uncorrected; that is, *p* < 1e − 6). This contrast was limited to voxels that were not significant (*z* > =3) in the “original versus scrambled” mask, to restrict EVC to voxels that were not in LOC. Additionally, the ROI mask was limited using an anatomical mask of the Harvard Oxford atlas, by removing voxels outside the occipital pole and the occipital fusiform gyrus. The resulting EVC mask contained V1–3 and V4 according to the Juelich Histological Atlas (Amunts et al., 2020).

### fMRI data analysis

In this study, we employed *FSL* 6.0.5 (FMRIB Software Library; Jenkinson, Beckmann, Behrens, Woolrich, & Smith, 2012; RRID:SCR_002823) interfaced through *Nipype* 1.8.2 (Gorgolewski et al., 2011; Gorgolewski et al., 2018; RRID:SCR_002502) using *Python* 3.10.5 to analyze the fMRI data.

For the functional images of each experimental run, we conducted spatial smoothing using SUSAN with a Gaussian kernel (full width at half maximum of 5 mm) and applied temporal high-pass filtering with a cutoff at 100 s. At the first level of analysis, we included 12 regressors of interest. These corresponded to the two stimulus types (convergent and divergent), each with six conditions (two occlusion, two repetition, and two alternation). Additionally, a separate regressor of no interest was incorporated, modeling the task trials. To account for further variations, we added the first temporal derivatives to the GLM. Our design matrix also integrated various confound regressors calculated via fMRIPrep: framewise displacement, six standard motion parameters, six anatomical CompCor components, two cosines, and motion outliers.

The contrasts of interest were defined as follows: (1) “mean activation,” encompassing all regressors of interest; (2) “convergent versus divergent,” indicating the BOLD response to convergent repetition and alternation trials, minus those of the divergent type; (3) “alternation versus repetition,” representing alternation minus repetition trials; (4) “structure effect,” signifying the BOLD response to occlusion trials following structural cues, minus those without such cues; and (5) “knowledge effect,” illustrating the BOLD response to occlusion trials following knowledge cues, minus those without such cues.

We employed voxel-wise GLMs fitted to individual participants’ run data using FSL FEAT in an event-related analysis. The events were represented as regressors with a duration of 1.2 s, encompassing the total duration of both the first and second stimuli in a trial, including the interstimulus interval. These regressors were convolved with a double gamma hemodynamic response function. Data across runs were combined through FSL’s Fixed Effects Analysis (FLAME), and data across participants were consolidated using FSL’s Mixed Effects Analysis (FLAME 1).

The outcomes of the mixed-effects analysis were directly utilized in the ROI analysis. Specifically, for each ROI, we computed the average BOLD response across all voxels within the designated mask and compared it across different conditions.

In addition to the ROI analysis, we conducted a whole-brain analysis by aggregating results across the entire participant population. To address the issue of multiple comparisons, we employed Gaussian random field cluster thresholding with a cluster formation threshold of *p* < 0.001 (one-sided; equivalent to *z* = 3.1) and a cluster significance threshold of *p* < 0.05. If not mentioned otherwise, all references to anatomical landmarks were based on the Harvard–Oxford Cortical Structural Atlas (Desikan et al., 2006).

### Eye movement analysis

During the fMRI session, we collected data from the left eye to investigate potential variations in eye movements or pupil dilation under different experimental conditions, as these variations could act as confounding factors in the actual analysis (Thielen et al., 2018; Thielen et al., 2019a).

To ensure the quality of the eye-tracking data, we used linear interpolation to handle missing data resulting from eye blinks, covering a time window from 100 ms before the blink onset to 100 ms after. Moreover, we applied spectral filtering with a low-pass cutoff set at 100 Hz to further enhance the data. Finally, the collected eye-tracking data were segmented into trials relative to the onset of the first stimulus until 1.2 s after, encompassing both stimuli presentation and the interstimulus interval.

For consistency and ease of analysis, we centered the *x* and *y* coordinates at the center of the screen (i.e., the point of fixation) and converted them to degrees of visual angle. To simplify the statistical analysis, we calculated trial averages, reducing each trial to a single scalar value for *x*, *y*, and pupil dilation.

### Data availability

The data that support the findings of this study are openly available through a data-sharing collection hosted on the Radboud Data Repository; see Thielen and Lier (2023). This collection comprises all raw data presented in the BIDS format, accompanied by the scripts and stimulus materials utilized for both the experimental procedures and data analysis.

## Results

### ROI definition

**Figure 4.**
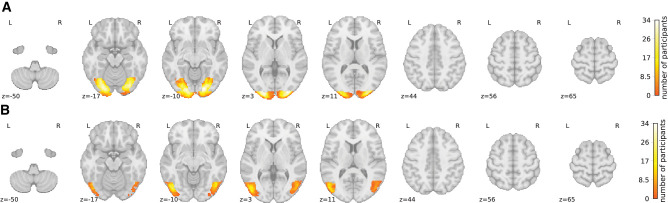
Functional ROI masks. The functional region of interest (ROI) masks for early visual cortex (EVC) (A) and lateral occipital complex (LOC) (B) as summed over participants, indicating for how many participants a voxel was contained in the mask. This visualization demonstrates that overall, there was an agreement of the location of the ROIs across participants, the EVC mask contains more voxels than the LOC mask, and the LOC mask seems to show a left-hemispheric dominance.

We set out an analysis to investigate the involvement of EVC and LOC in amodal completion. Participants each underwent a functional localizer to locate these two ROIs using the unoccluded objects from this study. Figure 4 depicts the overlap of these ROI masks over participants and shows that the ROI definitions are largely shared between participants.

For each ROI in both hemispheres, we determined the MNI coordinates (*x*, *y*, *z*) of the voxel that was most frequently contained within the mask, as detailed in Table 1. In the case of the left EVC, the predominant coordinates were (−27, −94, 4), with a range of 201 to 1,813 voxels in the mask. For the right EVC, the coordinates were (18, −87, −8), with about 170 to 1,645 voxels in the mask. This demonstrates that the EVC mask exhibited a balanced distribution across hemispheres, although it did vary among participants, averaging 2,180 voxels with a standard deviation of 709 for the full bilateral EVC mask.

**Table 1.** Functional ROI locations. The MNI coordinates (*x*, *y*, *z*) and number of voxels (minimum, maximum, average, standard deviation) of each of the regions of interest (ROIs): early visual cortex (EVC) and lateral occipital complex (LOC). Note, these MNI coordinates refer to the location that was most frequently found over participants in the ROI masks.

	MNI coordinates			Number of voxels				
Functional ROI	x	y	z		Min	Max	Avg	*SD*
EVC left	−27	−94	4		201	1,813	1,106	366
EVC right	18	−87	−8		170	1,645	1,073	363
EVC					371	3,435	2,180	709
LOC left	−49	−77	−2		71	914	417	235
LOC right	49	−71	−6		0	729	255	200
LOC					202	1,490	672	415

In contrast, for the left LOC, the MNI coordinates were (−49, −77, −2), with 71 to 914 voxels in the mask. The right LOC had coordinates of (49, −71, −6), with a total of 0 to 729 voxels in the mask. This revealed a left-hemisphere dominance and an overall smaller bilateral ROI when compared to the EVC, averaging 672 voxels with a standard deviation of 415 voxels.

### ROI analysis

**Figure 5.**
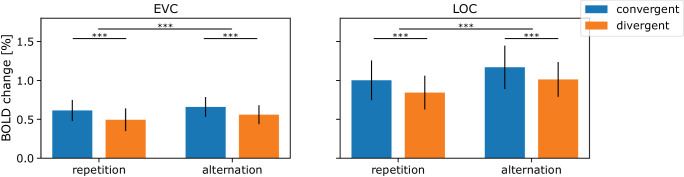
ROI analysis repetition suppression. The graph presents the grand average parameter estimates and their corresponding standard deviations for both stimulus types, convergent (blue) and divergent (orange), and both trial types, repetition and alternation. The analysis was performed in two regions of interest (ROIs): the early visual cortex (EVC, left) and the lateral occipital complex (LOC, right). In both the EVC and the LOC, a main effect of trial type was found, with responses in repetition trials to be significantly suppressed compared to those in alternation trials. This suggests that presenting the same stimulus consecutively led to a reduced neural response compared to when stimuli were presented in an alternating manner, the repetition suppression effect. Furthermore, across both ROIs, a main effect of stimulus type was found, with divergent stimuli eliciting significantly smaller neural responses than convergent stimuli, regardless of trial type. ***p < 0.001.

First, we investigated whether the repetition suppression effect of the fruit and vegetable stimuli was robustly present, by analyzing the difference in mean activation across ROIs and repetition and alternation trials (i.e., ignoring occlusion trials); see Figure 5. A repeated-measures analysis of variance (ANOVA) determined that the mean BOLD response varied significantly with trial type (EVC: *F*(1, 33) = 18.2552, *p* < 0.001; LOC: *F*(1, 33) = 139.1674, *p* < 0.001). A post hoc analysis showed that alternation trials (EVC: μ = 0.609, σ = 0.152; LOC: μ = 1.091, σ = 0.278) had a mean activation that was higher than repetition trials (EVC: μ = 0.554, σ = 0.174; LOC: μ = 0.922, σ = 0.280). These results confirm the classic repetition suppression effect where repeated presentation of the same stimulus leads to a reduced BOLD response in both ROIs.

The analysis also determined that the mean BOLD response varied significantly with stimulus type (EVC: *F*(1, 33) = 72.7788, *p* < 0.001; LOC: *F*(1, 33) = 91.3072, *p* < 0.001). A post hoc analysis showed that convergent stimuli (EVC: μ = 0.636, σ = 0.159; LOC: μ = 1.085, σ = 0.308) had a mean activation that was higher than divergent stimuli (EVC: μ = 0.527, σ = 0.153; LOC: μ = 0.928, σ = 0.250). There results show that there is an overall lower BOLD response for divergent stimuli than for convergent stimuli, regardless of trial type as well as ROI.

There was no interaction found between trial type and stimulus type (EVC: *F*(1, 33) = 0.9201, *p* = 0.344; LOC: *F*(1, 33) = 0.0054, *p* = 0.942).

**Figure 6.**
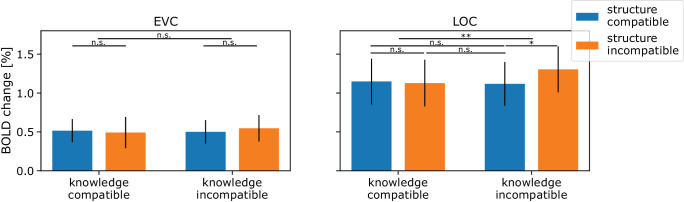
ROI analysis occlusion. The graph shows the grand average parameter estimates and their corresponding standard deviations for occlusion trials categorized as structure compatible (S+, blue) and structure incompatible (S−, orange), as well as knowledge compatible (K+) and knowledge incompatible (K−) completions. Two regions of interest (ROIs) were analyzed: the early visual cortex (EVC, left) and the lateral occipital complex (LOC, right). In EVC, none of the observed differences between the conditions were found to be statistically significant. In LOC, responses were significantly suppressed in response to knowledge compatible completions as compared to knowledge-incompatible completions. Additionally, in LOC, completions that were structure compatible were suppressed more than those incompatible with structure. n.s. = not significant. *p < 0.05. **p < 0.01.

Second, we investigated whether the repetition suppression effect of the fruits and vegetables varies within the occlusion trials, each following different cues of structure and knowledge; see Figure 6. A repeated-measures ANOVA determined that the mean BOLD response varied significantly with structure (i.e., completable according to linear continuation or not) in LOC (*F*(1, 33) = 11.4093, *p* = 0.002), but not in EVC (*F*(1, 33) = 0.4172, *p* = 0.523). A post hoc analysis demonstrated that trials compatible with structure (μ = 1.133, σ = 0.291) showed lower activation than those incompatible with structure (μ = 1.215, σ = 0.312) in LOC but not in EVC (structure compatible: μ = 0.508, σ = 0.152, structure incompatible: μ = 0.519, σ = 0.189). Following the rationale of repetition suppression where repeated presentation of the same stimulus leads to a reduction in the BOLD response, these results show that in LOC, the completion compatible with a linear continuation is preferred, while in EVC, this is not the case.

The analysis also determined that the mean BOLD response varied significantly with knowledge (i.e., completable according to familiarity or not) in LOC (*F*(1, 33) = 9.7273, *p* = 0.004), but again not in EVC (*F*(1, 33) = 1.0446, *p* = 0.314). A post hoc analysis demonstrated that those completions that were compatible with knowledge (μ = 1.138, σ = 0.300) showed lower activation than the completions that were incompatible with knowledge (μ = 1.211, σ = 0.300) in LOC but not in EVC (knowledge compatible: μ = 0.503, σ = 0.179; knowledge incompatible: μ = 0.524, σ = 0.164). Similarly, these results suggest that there is a preference for completions compatible with the expected shape given our prior knowledge, but again only in LOC but not in EVC.

There was an interaction found between structure and knowledge in LOC (*F*(1, 33) = 19.2325, *p* < 0.001). Specifically, structure-compatible but knowledge-incompatible occlusion trials showed the lowest BOLD response (S+K−: μ = 1.118, σ = 0.286), followed by structure-incompatible but knowledge-compatible occlusion trials (S−K+: μ = 1.127, σ = 0.305) and structure- and knowledge-compatible trials (S+K+: μ = 1.148, σ = 0.299), and the highest BOLD response was found for structure- and knowledge-incompatible occlusion trials (S−K−: μ = 1.304, σ = 0.298). These results affirm that completions that deviate from expectations rooted in both structure or knowledge (S−K−) resulted in the least reduction in the BOLD response within the LOC. Indeed, in all pairwise comparisons, a significant difference was found with S−K− (S+K+ *p* = 0.036, S+K− *p* = 0.011, S−K+ *p* = 0.019). Surprisingly, completions that aligned with both expectations (S+K+) did not exhibit a more substantial reduction than cases where only one cue was incompatible (S+K− or S−K+). Specifically, none of the pairwise comparisons between S+K+, S+K−, or S−K+ were significantly different (*p* > 0.600).

### Whole-brain analysis

**Figure 7.**
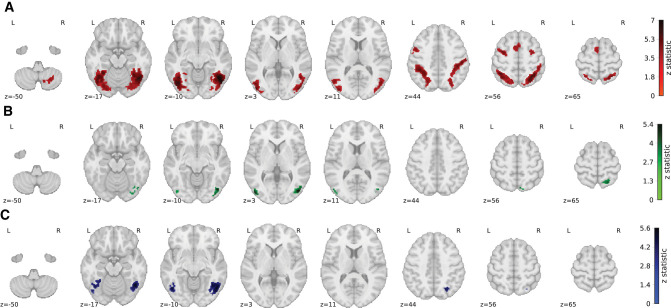
Whole-brain analysis. The whole-brain exploratory cluster analysis for (A) the repetition suppression effect where the contrast is defined as alternation trials minus repetition trials (red), (B) the structure effect where the contrast is defined as occlusion trials incompatible with structure (S−) minus structure compatible (S+) (green), and (C) occlusion trials incompatible with knowledge (K−) minus knowledge compatible (K+) (blue). Note that for the reversed effects (repetition minus alternation, structure compatible minus incompatible, and knowledge compatible minus incompatible), no significant clusters were found. Overall, these results show a clear repetition suppression effect and modulation of structure and knowledge in the higher-order visual ventral stream.

We carried out an exploratory whole-brain analysis to explore potentially interesting effects outside the two ROIs as defined before; see Figure 7. Importantly, the cluster analysis was able to identify significant clusters only for the contrasts alternation minus repetition, structure incompatible minus structure compatible, and knowledge incompatible minus knowledge compatible, not their reversed counterparts.

First, from the significant clusters in the classic repetition suppression contrast (alternation minus repetition; Figure 7A), several observations can be made. (1) EVC did not respond significantly different to the two types of trials. (2) Both the inferior (ventral, left (−41, −71, −11), right (45, −61, −9)) as well as the superior (dorsal, left (−35, −50, 48), right (28, −59, 58)) part of LOC showed a significant difference. Additionally, (3) also three frontal areas were identified (left (−47, 3, 48), central (0, 9, 56), and right (28, −3, 50)), located in the middle frontal gyrus and precentral gyrus, with each involved in higher cognitive abilities such as attention and working memory. Finally, (4) one cluster was found in right cerebellum (31, −63, −53), which might be implicated due to sensory-motor integration of the naturalistic objects used in the paradigm.

Second, regarding the structure cue in occlusion trials, we investigated the contrast between structure-incompatible minus structure-compatible trials. Here we found clusters in left inferior (ventral) LOC (−47, −83, 2) as well as right inferior (ventral) LOC (47, −79, 4), and finally a cluster in the superior (dorsal) LOC toward the superior parietal lobule (22, −60, 65).

Third, regarding the knowledge cue in occlusion trials, we investigated the contrast between knowledge-incompatible minus knowledge-compatible trials. Here we found clusters in left inferior (ventral) LOC (−47, −65, −9) as well as right inferior (ventral) LOC (47, −57, −17), and finally a cluster in superior (dorsal) LOC (26, −67, 44). Of interest is the observation that the knowledge clusters seem to be more anterior as well as more ventral relative to the structure clusters that are more posterior and more dorsal.

The whole-brain analysis suggested a prevailing dominance in the right hemisphere for both structural and knowledge-related contrasts. To validate these findings, we conducted further examinations by creating a 10-mm sphere centered on the voxel with the highest amplitude within a cluster. We then extracted the mean BOLD response within this sphere across all participants. Subsequently, we subjected these mean values to a paired one-sided *t*-test to assess whether the right hemisphere exhibited greater activity compared to the left hemisphere. The analysis of the structural contrast revealed no significant right-hemispheric dominance (*t* = −0.048, *p* = 0.519). However, in the case of the knowledge-related cluster, we observed a statistically significant right-dominant lateralization (*t* = 1.793, *p* = 0.041).

### Task performance and eye movement analysis

During the main task of the experiment, participants engaged in a series of trials that involved the presentation of two stimuli sequentially. On various occasions, they performed a 1-back task, wherein a statement regarding the two previously presented stimuli was provided, and participants had to determine whether the statement was correct or incorrect. The accuracy of this task varied between a minimum of 50% and a maximum of 100% (μ = 81.3%, σ = 11.4%). These results indicate that the task was feasible, yet challenging for the participants.

**Figure 8.**
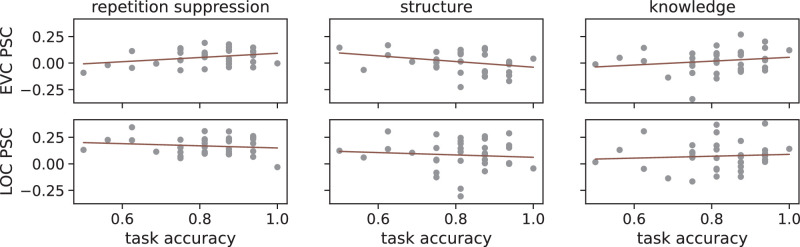
Task performance. Depicted are the relationships between the task performance of individual participants and their three main contrasts (alternation minus repetition, structure incompatible minus compatible, knowledge incompatible minus compatible; see Figure 7), for which the average percent of signal change (PSC) of the neural response for each of the regions of interest (ROIs) is shown: early visual cortex (EVC) and lateral occipital complex (LOC). The orange lines show an ordinary least squares regression. According to the Pearson’s correlation coefficient, none of these linear relationships were significant (*p* > 0.05), meaning that none of the neural contrasts could be explained by participants’ task performance.

Because attention has been shown to affect repetition suppression, we performed post hoc correlation analyses to assess whether the task performance varies with the repetition suppression and the suppression effects in the occlusion trials in this study; see Figure 8. For both ROIs, we calculated the Pearson’s correlation coefficient *r* between task performance and the average BOLD contrast of alternation minus repetition trials (EVC: *r* = 0.302, *p* = 0.082; LOC: *r* = −0.139, *p* = 0.434), task performance and the average BOLD contrast of structure-incompatible minus structure-compatible trials (EVC: *r* = −0.299, *p* = 0.085; LOC: *r* = −0.089, *p* = 0.616), and task performance and the average BOLD contrast of knowledge-incompatible minus knowledge-compatible trials (EVC: *r* = 0.180, *p* = 0.310; LOC: *r* = 0.074, *p* = 0.676). These results indicate that none of the contrasts could be explained by the task performance.

We examined the eye-tracking data to determine whether eye movements or pupil dilation could account for the aforementioned results. Specifically, we conducted separate repeated-measures ANOVAs for each of the *x*, *y*, and pupil dilation measurements, employing the same two-factor designs as used for studying the BOLD responses. Most of these analyses yielded insignificant results (*p* > 0.05), except for a significant main effect of structure on the *x* position (*F*(1, 32) = 10.4374, *p* = 0.003). A post hoc analysis revealed that in occlusion trials, the *x* position was larger for those compatible with structure (μ = 0.18, σ = 2.29) than for those incompatible with structure (μ = 0.10, σ = 2.30).

## Discussion

In this study, we used a repetition suppression paradigm to investigate BOLD responses using familiar naturalistic objects. Following an ROI analysis, we successfully observed a pronounced repetition suppression effect in both the EVC and the LOC, demonstrated by a diminished response to repeated stimuli compared to alternating stimulus presentations. Notably, our stimulus repertoire encompassed partially occluded objects, where a relatively higher repetition suppression was expected (i.e., a lower BOLD response) if the perceptually completed object coincided with the subsequent presentation. In this study, we examined two cues for perceptual completion defined as compatibility with structure (linear continuation) and/or knowledge (familiarity), thereby dissociating their influences. While no discernible differences emerged among these conditions in EVC, LOC exhibited a significant impact of both structure and knowledge. Here, the BOLD response was relatively increased (with reduced repetition suppression) for completions incongruent with both structure and knowledge, in comparison to the other conditions.

Repetition suppression pertains to the decline in neural activity upon stimulus repetition. This phenomenon is attributed to the fatiguing of neurons attuned to the repeated stimulus or a more effective encoding of subsequent presentations, leading to a refinement of the recruited neuron population (Grill-Spector & Malach, 2001; Grill-Spector, Henson, & Martin, 2006). The manifestation of this effect aligns with the observed main effect of structure within LOC and shows that the repetition suppression paradigm works well to study amodal completion, as was also employed by several other studies (Rauschenberger et al., 2006; Weigelt et al., 2007).

Conversely, expectation suppression stems from top-down perceptual expectations. This suppression arises when neural activity reflects a reduction in the perceptual prediction error, predominantly when sensory evidence aligns with a more probable (e.g., previously encountered) perception, compared to a less likely (e.g., novel) one (Summerfield, Trittschuh, Monti, Mesulam, & Egner, 2008; Richter & de Lange, 2019). This finding parallels the observed effect of knowledge within LOC, illustrating that the least repetition suppression transpired in conditions that defied both structural expectations and knowledge, thus forming the least expected completion.

It is noteworthy that expectation suppression is contingent on attention. Diverting attention away from a stimulus diminishes the role of expectation in shaping neural activity (Larsson & Smith, 2012; Richter, Ekman, & de Lange, 2018). In our study, participants were instructed to attend to the presented stimuli to execute the 1-back task effectively. A post hoc analysis of task performance against BOLD percent signal change did not yield conclusive evidence for the involvement of attention in the observed contrasts. However, one should be careful with the interpretation of such correlation analysis as it might require a larger sample size. A follow-up investigation might explore whether our findings persist under conditions where participants perform a demanding task at fixation, thereby diverting attention away from stimuli. Particularly, amodal completion is deemed an automatic process independent of conscious thought, suggesting that repetition suppression might still occur for amodally completed objects even without attention, in contrast to the highlighted attention-mediated effect on repetition suppression. It would also be particularly interesting to see whether the familiarity effect that we observed in this study would still stand when attention is diverted away.

The absence of a distinct amodal completion signature in EVC resonates with prior studies that also did not detect activity in response to partially occluded objects within this region (Lerner et al., 2002; Lerner et al., 2004; Olson et al., 2004; Weigelt et al., 2007; Rampone, Adam, Makin, Tyson-Carr, & Bertamini, 2022). Nonetheless, other investigations did report EVC involvement (Rauschenberger et al., 2006; Hulme & Zeki, 2007; Ban et al., 2013; Erlikhman & Caplovitz, 2017; de Haas & Schwarzkopf, 2018). The present study contributes to the growing evidence that amodal completion is a multifaceted phenomenon, contingent upon stimulus intricacy and salience, thus eliciting a diverse array of observed neural patterns (Thielen et al., 2019b). Specifically, in the current study, we used naturalistic stimuli, while those studies that did show EVC involvement predominantly used simple lines or line-drawing stimuli (Sugita, 1999; Lerner et al., 2002; Rauschenberger et al., 2006).

Intriguingly, our study revealed that completions inconsistent with both structural and knowledge-based expectations (S−K−) exhibited the least suppression, aligning with findings from Hazenberg and van Lier (2016). These results are in accordance with the notion of expectation suppression, suggesting that the brain forms expectations, here based on structural and knowledge cues, resulting in reduced and more efficient processing when subsequent stimuli align with these expectations. However, this would also imply that completions compatible with both structure and knowledge (S+K+) should lead to a further reduction in the BOLD response compared to completions where one of the cues is incompatible (S+K− or S−K+), as observed by Hazenberg and van Lier (2016). Interestingly, for unknown reasons, this particular effect was not observed in our current study.

In addition to the ROI analysis, we conducted a whole-brain analysis, which confirmed a prominent repetition suppression effect throughout the extra-striate cortex, consistent with findings from the ROI analysis. Interestingly, unlike the ROI analysis, the striate cortex did not feature prominently in this analysis, possibly due to the application of a more conservative cluster-based statistical thresholding method in the whole-brain analysis. The fact that EVC was already absent in the classic repetition suppression effect precludes the study of any involvement of structure and knowledge cues in EVC. Moreover, the whole-brain analysis unveiled several additional clusters outside the ROIs. Specifically, in addition to both the ventral and dorsal LOC, three frontal clusters emerged. These frontal regions are typically associated with repetition suppression and linked to working memory, as documented in previous studies (Segaert, Weber, de Lange, Petersson, & Hagoort, 2013; Richter & de Lange, 2019). However, it is worth mentioning that these frontal clusters were not identified when contrasting compatibility with the structure and knowledge cues in occlusion trials.

Notably, our whole-brain findings revealed a distinct asymmetry in the processing of naturalistic fruit and vegetable stimuli in three significant ways. First, a left-hemispheric dominance was evident within the ROI masks for the LOC. This mask, derived from a functional localizer based on fully visible (nonoccluded) fruit and vegetable stimuli, may be primarily associated with general object perception, rather than with amodal completion specifically. These findings diverge from the conventional pattern where object perception effects are primarily associated with right-hemispheric dominance, as seen, for instance, in face perception localized in the fusiform face area (Kanwisher, McDermott, & Chun, 1997). Notably, in scene perception, lateralization effects have been observed in both the left and right hemispheres, contingent upon factors such as local or global processing and the nature of the stimulus category (Fink et al., 1997). Second, our whole-brain analysis highlighted a right-hemispheric dominance, particularly in the processing of the knowledge cue. These results align with a previous study involving two callosotomy patients, which suggested that amodal completion relies more on higher-level visual processing and the right hemisphere in contrast to modal completion, which depends more on early visual processing occurring in both hemispheres (Corballis, Fendrich, Shapley, & Gazzaniga, 1999). Within a post hoc analysis, we did not find evidence for a difference in temporal signal-to-noise ratio (tSNR) between the two hemispheres (left: μ = 45.2, σ = 6.1; right: μ = 44.0, σ = 11.5; *p* = 0.511) that could account for these results. Finally, in line with the results of the ROI analysis, the whole-brain analysis revealed significant clusters in bilateral LOC for both the structure and knowledge cues, and it also showed clusters in both ventral and dorsal parts of the LOC. Interestingly, the clusters related to knowledge were found more anterior as well as more ventral relative to the clusters related to structure, showing a potentially higher reliance on association cortex.

It is essential to approach the results of our study on amodal completion with caution and consider several important limitations. First, the ROI analysis indicated a statistical difference between the convergent and divergent stimulus sets. These distinctions could potentially be attributed to variations in luminance, color, and shape between the two sets. It is crucial to emphasize that our study did not specifically aim to investigate such main effects of stimulus type but rather focused on the interaction between structure and knowledge cues across these sets. Therefore, no direct contrast between convergent and divergent stimuli was conducted.

Second, a post hoc analysis of participants’ eye movements revealed a significant difference in horizontal eye movements when they were presented with occlusion trials that were either compatible or incompatible with the underlying structure cue. Although the average difference in horizontal eye position was relatively small (0.08 degrees of visual angle), future research should explore potential alterations in eye movement dynamics relative to the type of completion. Additionally, further research might investigate why such eye movements emerge, which potentially could have a link with completing an object as one whole, or as two parts separated by empty space behind the occluder.

Third, the terms “structure” and “knowledge” were employed in a relatively loose manner in our study. The structure cue was primarily associated with rectilinear continuations, but low-level structural cues could also pertain to curvilinear continuations, which might come into play when completing the outline of objects such as apples. Similarly, the knowledge cue was linked to the familiarity of objects, with elongated apples and oranges clearly deviating from natural forms. However, small bananas and cucumbers may still fall within the realm of relatively natural and thus familiar shapes, more so than elongated apples or lemons. These observations are in line with familiarity ratings from participants in the study by Hazenberg and van Lier (2016). They showed that while both knowledge-incompatible completions were rated as being rather strange, completions compatible with structure (i.e., elongated apples) were rated as being stranger relative to completions incompatible with structure (i.e., small bananas). Given these ratings it is interesting to note that when looking at the BOLD responses in the current study, it is not the (behaviorally assessed) “strangest” completion that stands out. Instead, completions that are a little less strange elicited results that deviated from the results that were elicited by the other completions. This shows that the BOLD responses are not just driven by perceived familiarity but that structure plays a crucial role as well.

Fourth, our study employed broadly defined ROIs. The EVC mask encompassed regions beyond V1–3, including also V4 according to the Juelich Histological Atlas (Amunts, Mohlberg, Bludau, & Zilles, 2020). Similarly, the LOC mask was anticipated to involve both ventral and lateral components, including LO1 and LO2 (Larsson & Heeger, 2006). Follow-up studies could further explore the nuanced contributions of subregions within the expansively defined masks used in this study, shedding light on their individual and potentially distinct roles in amodal completion.

In light of these considerations, our study provides valuable insights into amodal completion, but further research and replication are warranted to enhance our understanding of the complex interactions between structure and knowledge cues in visual perception.

## Conclusions

In this study, we investigated amodal completion using naturalistic objects within a repetition suppression paradigm while distinguishing between structure and knowledge cues. The lateral occipital complex displayed differential responses based on both structural and knowledge cues. Furthermore, we observed an interaction where the suppression effect was minimized when the stimulus was incompatible with both structure and knowledge cues. In early visual cortex, we did not not find evidence for differential responses to completions that were compatible relative to those incompatible with the underlying structure. Similarly, in the early visual cortex, we did not find evidence for varied responses to completions that were compatible with knowledge compared to those that were incompatible. Together, our results suggest that the process of amodal completion is not only influenced by structure and knowledge cues, but also that both these two cues are primarily prominent in higher-level visual areas, with less impact on the early visual cortex.
